# Correlation of hypogammaglobulinaemia with proteinuria, and the relationship between hypogammaglobulinaemia and infection in active lupus nephritis

**DOI:** 10.1136/lupus-2017-000229

**Published:** 2017-08-31

**Authors:** Dawn Elaine Smilek, Noha Lim, Linna Ding, Sara G. Murray, Betty Diamond, David Wofsy

**Affiliations:** 1 Immune Tolerance Network, University of California San Franciso, San Francisco, California, USA; 2 Division of Rheumatology, Department of Medicine, and the Lupus Nephritis Trials Network, University of California San Francisco, San Francisco, California, USA; 3 Immune Tolerance Network, Massachusetts General Hospital, Bethesda, Maryland, USA; 4 Division of Allergy, Immunology, and Transplantation, National Institute of Allergy and Infectious Diseases, Rockville, Maryland, USA; 5 Division of Hospital Medicine, Department of Medicine, University of California San Francisco, San Francisco, California, USA; 6 Center for Autoimmune and Musculoskeletal Diseases, The Feinstein Institute for Medical Research, Manhasset, New York, USA; 7 Division of Rheumatology, Department of Medicine, and the Russell/Engleman Research Center, University of California San Francisco, San Francisco, California, USA

**Keywords:** Cyclophosphamide, Infections, Lupus Nephritis

## Abstract

**Objective:**

To evaluate hypogammaglobulinaemia and risk of serious infectious adverse events in active lupus nephritis.

**Methods:**

The Abatacept and Cyclophosphamide Combination Efficacy and Safety Study (ACCESS) compared abatacept with placebo in participants with lupus nephritis undergoing treatment with Euro-Lupus Nephritis low-dose cyclophosphamide. Serum IgG levels were assessed prior to initiation of treatment and throughout the trial. Hypogammaglobulinaemia was defined as IgG <450 mg/dL.

**Results:**

Hypogammaglobulinaemia was observed in 16/102 (15.7%) participants prior to initiation of induction therapy for active lupus nephritis. Participants with nephrotic range proteinuria were more likely to have hypogammaglobulinaemia, and serum IgG levels were inversely correlated with urine protein to creatinine ratio (r=−0.42, p<0.0001). Following initiation of treatment for active lupus nephritis, additional participants developed hypogammaglobulinaemia by weeks 2–4. Serum IgG levels then increased, and all but one participant had serum IgG ≥450 mg/dL at 24 weeks. Hypogammaglobulinaemia was not associated with an increased risk of serious infectious adverse events.

**Conclusions:**

In active lupus nephritis in ACCESS, hypogammaglobulinaemia was common and inversely correlated with proteinuria. Serum IgG levels were lowest in the weeks immediately following initiation of induction therapy, and subsequently improved by 24 weeks. Hypogammaglobulinaemia was not associated with serious infectious adverse events.

**Trial registration:**

## Introduction

Reduced serum IgG has been reported in lupus nephritis (LN)^1 2^; however, levels are not routinely measured, and the prevalence of hypogammaglobulinaemia in active LN is not known. Persistent trough levels of IgG below 400 mg/dL in common variable immunodeficiency and X linked agammaglobulinaemia have been associated with increased risk of infection, pneumonia in particular,^3^ but there is little evidence that hypogammaglobulinaemia in active LN confers a similar risk. However, a phase II/III trial with atacicept in LN was halted after enrolment of six participants, when severe infections occurred in two participants with hypogammaglobulinaemia.^4^ Therefore, evaluation of serum IgG levels during treatment for active LN was undertaken in the Abatacept and Cyclophosphamide Combination Efficacy and Safety Study (ACCESS)^5^ to evaluate the relationship of serum IgG levels to serious infectious adverse events (SIAEs).

## Patients and methods

### Study treatment

Details of ACCESS have been previously described.^5^ Briefly, ACCESS was a multicentre randomised controlled trial of abatacept compared with placebo in 134 participants with LN who underwent treatment with the Euro-Lupus Nephritis (ELN) low-dose cyclophosphamide regimen^6^ followed by azathioprine maintenance therapy. Participants also received prednisone. ACCESS, registered as NCT00774852, received institutional review or ethics board approval at each site, and was conducted in accordance with the International Conference on Harmonisation Guidelines for Good Clinical Practice and the Declaration of Helsinki.

### Assessments

Quantitative serum IgG assessments were instituted after the initiation of ACCESS, and were assessed at the screening visit and at weeks 0, 2, 4, 12, 24 and 52. Hypogammaglobulinaemia was defined as serum IgG <450 mg/dL since serum IgG was expected to decline in the setting of LN treatment, and the risk for infection in immunodeficiency syndromes is associated with serum IgG <400 mg/dL.^3^ Nephrotic range proteinuria was defined as urinary protein ≥3.5 g in a 24-hour urine collection. Adverse events (AEs) were graded according to the National Cancer Institute Common Terminology Criteria for Adverse Events v3.0 (NCI CTCAE v3.0).

### Statistical methods

Comparisons of categorical variables were performed using a Χ^2^ test or a Fisher exact test depending on the cell sizes. Comparisons of continuous variables were performed using a Wilcoxon rank-sum test. Pearson’s correlation coefficient was calculated to determine associations between IgG level and urine protein to creatinine ratio (UPCR). All statistical tests were two-sided.

## Results

### Hypogammaglobulinaemia in active LN correlated with proteinuria

In ACCESS, 134 participants were treated for active LN with the ELN cyclophosphamide induction regimen and prednisone, in combination with abatacept or placebo. Serum IgG levels were examined beginning at the screening visit in 102 participants. Hypogammaglobulinaemia was observed in 16/102 (15.7%) participants prior to induction therapy. Four participants had serum IgG <300 mg/dL, and the lowest level of IgG observed prior to induction therapy was 170 mg/dL. Table 1 shows the characteristics of 16 participants with IgG <450 mg/dL prior to induction therapy, compared with 86 participants with IgG ≥450 mg/dL prior to induction therapy.

**Table 1:** Characteristics of participants with hypogammaglobulinaemia prior to induction therapy for lupus nephritis

	IgG <450 mg/dL	IgG ≥450 mg/dL	p Value
	n=16	n=86	
Age	29.8±7.2	32.3±11.0	0.61
Gender			0.35
Female	16/16 (100%)	77/86 (90%)	
Race			0.51
Asian	1/16 (6%)	2/86 (2%)	
Black	8/16 (50%)	36/86 (42%)	
White	0/16 (0%)	6/86 (7%)	
Other	7/16 (44%)	42/86 (49%)	
Ethnicity			0.42
Hispanic or Latino	8/16 (50%)	53/86 (62%)	
Urinary protein ≥3.5 g*	13/16 (81%)	31/86 (36%)	<0.01
Lymphocyte count <1000	8/16 (50%)	43/85 (51%)†	>0.99
Lupus nephritis ≥12 months	8/16 (50%)	20/86 (23%)	0.04
MMF within past 30 days	4/16 (25%)	9/86 (10%)	0.12
CS within past 30 days	12/16 (75%)	62/86 (72%)	>0.99

*In a 24-hour urine collection.

†1 missing.

CS, corticosteroids; MMF, mycophenolate mofetil/mycophenolic acid.

Gender, age, race and ethnicity did not significantly differ between the two groups, nor did the proportion of participants with total lymphocyte counts <1000/µL. Mycophenolate mofetil and corticosteroid usage in the 30 days prior to enrolment was not significantly different. Participants did not receive cyclophosphamide or rituximab in the year prior to enrolment, or belimumab in the 6 months prior to enrolment.

**Figure 1:**
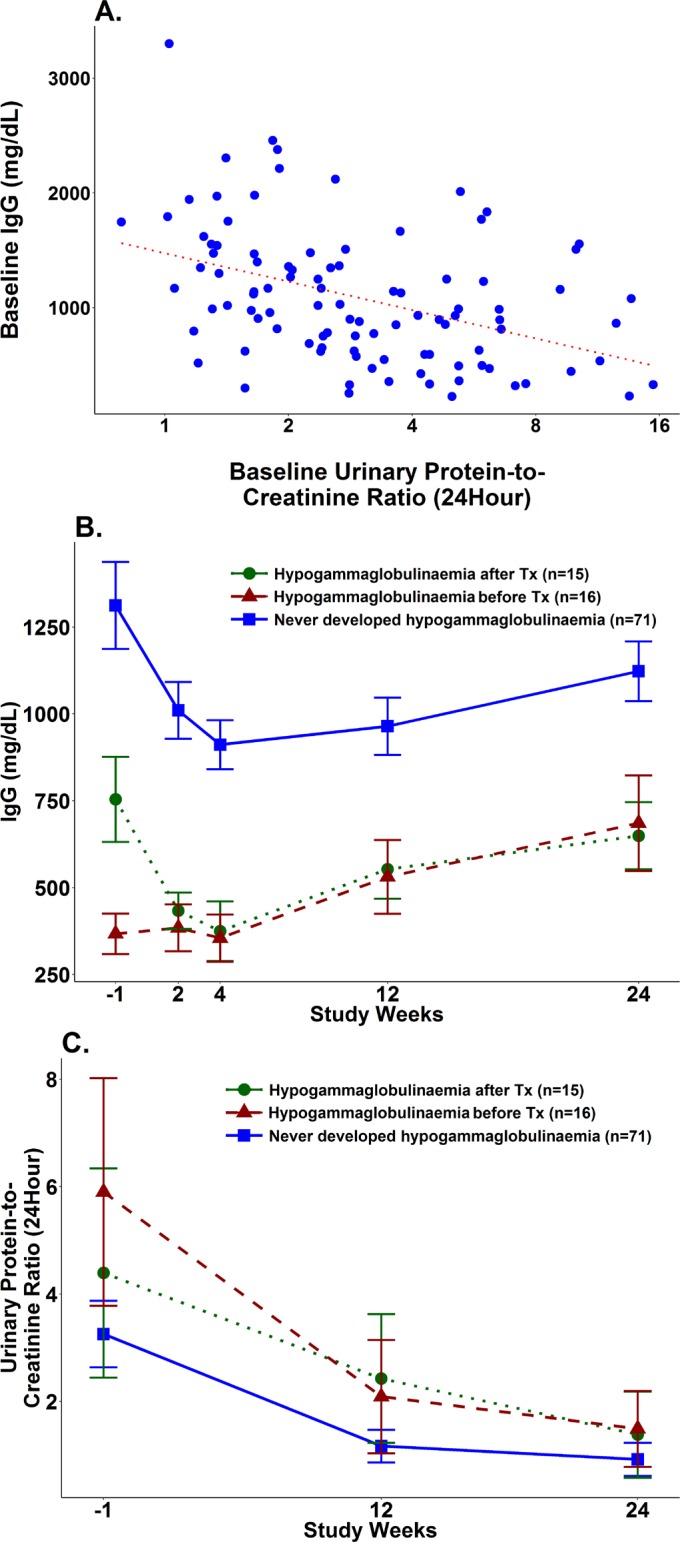
(A) Correlation between serum IgG and UPCR in a 24-hour collection. (B) Serum IgG levels before, during and after Tx. (C) UPCR in a 24-hour collection before, during and after Tx. Tx, induction therapy; UPCR, urine protein to creatinine ratio.

In contrast, participants with low serum IgG level prior to induction therapy were significantly more likely to have nephrotic range proteinuria compared with participants without low serum IgG (p<0.01, table 1). Hypogammaglobulinaemia was observed in 13/44 (30%) participants with nephrotic range proteinuria ≥3.5 g prior to induction therapy, compared with 3/58 (5%) without nephrotic range proteinuria. Hypogammaglobulinaemia prior to induction therapy was observed most frequently in the setting of high levels of proteinuria, occurring in 5/12 (42%) participants with proteinuria >8.0 g and in 3/4 (75%) participants with proteinuria >14 g in a 24-hour collection. Pearson’s correlation analysis showed an inverse correlation between IgG level and UPCR in a 24-hour collection (r=−0.42, p<0.0001, figure 1A). Low serum IgG prior to induction therapy was also associated with LN duration ≥12 months (p=0.04, table 1).

### Serum IgG levels initially decreased following treatment for active LN, then increased by 24 weeks

Low serum IgG levels prior to induction therapy remained low during the initial few weeks of treatment. Furthermore, serum IgG levels decreased following induction therapy in participants without hypogammaglobulinaemia prior to treatment (figure 1B). Fifteen additional participants developed hypogammaglobulinaemia following initiation of induction therapy, including five who developed IgG <300 mg/dL. Thus, hypogammaglobulinaemia was observed in a total of 31/102 (30.4%) participants, either prior to treatment or in the weeks immediately following treatment initiation, while 71/102 (69.6%) never developed hypogammaglobulinaemia. Similar proportions of participants with hypogammaglobulinaemia were observed in the abatacept group and the placebo group (data not shown).

Serum IgG levels tended to be lowest at week 2 or week 4, and then increased by week 12, whether hypogammaglobulinaemia was present prior to treatment, developed after treatment or never occurred (figure 1B). At study week 24, hypogammaglobulinaemia resolved to IgG ≥450 mg/dL in nearly all participants remaining in the study, corresponding to overall improvement in UPCR in all groups (figure 1C). Only one participant had residual low serum IgG of 380 mg/dL at week 24 and also had a poor renal response to therapy. At study week 52, all participants remaining in the study had serum IgG ≥450 mg/dL (data not shown).

### Hypogammaglobulinaemia was not associated with SIAEs

Eleven SIAEs occurred in nine participants during the 24 weeks of treatment (table 2).

**Table 2:** Serious infectious adverse events during the first 24 weeks of the ACCESS trial

Participant	Adverse event term	Bacterial	Start	Stop	Screening	Nadir
			Day	Day	IgG mg/dL	IgG mg/dL
1	Tubo-ovarian abscess	Yes	45	57	1470	1140
2	Cellulitis	Yes	1	6	630	431
3	Urinary tract infection	Yes	0	5	493	493
3	Pneumonia	Yes	5	51	493	493
4	Gastroenteritis	No	88	94	1554	1025
5	Bacteraemia	Yes	56	59	1268	1268
6	Viral infection	No	59	63	1143	797
7	Pneumonia	Yes	7	11	852	690
8	Gastroenteritis viral	No	95	110	577	347
9	Viral infection	No	38	41	688	327
9	Fever	Unknown	116	118	688	327

ACCESS, Abatacept and Cyclophosphamide Combination Efficacy and Safety Study.

Infectious AEs were considered serious if they were grade 3 or higher (according to the NCI CTCAE v3.0), or if the event resulted in or extended hospitalisation. SIAEs occurred in 3/31 participants who developed hypogammaglobulinaemia during the study, compared with 6/71 participants without hypogammaglobulinaemia (9.7% vs 8.4%). None of the SIAEs occurred in participants with hypogammaglobulinaemia at screening.

Of the 11 SIAEs that occurred, at least 6 were bacterial in nature. Cellulitis occurred the day following the first dose of study medication in one participant with IgG 630 mg/dL at the screening visit, who later developed IgG 431 mg/dL at week 4. The other bacterial infections occurred in participants in whom hypogammaglobulinaemia was never observed (table 2). No participants with hypogammaglobulinaemia withdrew from ACCESS due to an infectious AE. One participant with IgG 270 mg/dL at week 4 was withdrawn from the study and received intravenous immunoglobulin at the discretion of the site investigator, but no infectious AE occurred.

## Discussion

In 102 ACCESS participants with LN, hypogammaglobulinaemia was observed in nearly a third but was not associated with an increase in serious bacterial or other infections. Serum IgG correlated inversely with UPCR in a 24-hour collection, and participants with nephrotic range proteinuria were more likely to have serum IgG <450 mg/dL prior to initiation of induction therapy. Furthermore, hypogammaglobulinaemia resolved with treatment of the active LN in all but one participant.

Factors other than urinary loss likely contribute to low serum IgG in active LN. Hypogammaglobulinaemia was significantly more common in participants with LN of ≥12 months’ duration. Certain medications, including corticosteroids, are reported to cause secondary hypogammaglobulinaemia.^7 8^ In ACCESS, recent treatment with corticosteroids or mycophenolate mofetil in the prior 30 days was not associated with hypogammaglobulinaemia at study entry. However, serum IgG levels dropped following induction therapy with low-dose cyclophosphamide and prednisone, and then gradually rose, such that hypogammaglobulinaemia resolved in nearly all participants by 24 weeks. A similar result was observed in another study of IgG levels in LN, in participants treated with methylprednisolone and mycophenolate mofetil.^2^

Persistent hypogammaglobulinaemia in common variable immunodeficiency and X linked agammaglobulinaemia is associated with risk for serious infections.^3^ Sustained very low levels of IgG <100 mg/dL are associated with the highest risk, while transient or less severe hypogammaglobulinaemia is tolerated in most subjects.^7^ Transient hypogammaglobulinaemia is also generally well-tolerated in the setting of autoimmune disease.^8^ A retrospective study of rituximab-associated hypogammaglobulinaemia in multisystem autoimmune disease showed that hypogammaglobulinaemia occurred in 56% of 243 subjects treated with rituximab, and IgG replacement was undertaken in only 4.2% of subjects due to recurrent infection.^9 10^ In contrast, recurrent infections have been associated with hypogammaglobulinaemia in smaller vasculitis series,^11 12^ and a phase II/III trial with atacicept in LN was halted when severe infections occurred in the setting of hypogammaglobulinaemia.^4^

The ACCESS results demonstrate that hypogammaglobulinaemia in active LN is common, transient and well-tolerated. Most participants with hypogammaglobulinaemia in ACCESS did not develop serious infections, although a small increased risk of infection might not have been detected. The results are consistent with intact production of IgG despite urinary loss, and the adequate humoral response to vaccination observed in SLE.^13^

A number of factors can contribute to hypogammaglobulinaemia in LN, including proteinuria, corticosteroids and other immunosuppressive medications. Comorbid common variable immunodeficiency can also occur.^14 15^ Although these other factors should be considered, treatment of LN with induction therapy should not be delayed due to hypogammaglobulinaemia alone, and in ACCESS induction therapy for LN was followed by resolution of hypogammaglobulinaemia in nearly all participants. The ACCESS results do not support routine prophylactic treatment with replacement immunoglobulin for transient asymptomatic hypogammaglobulinaemia that occurs in LN, nor do they support exclusion of participants with hypogammaglobulinaemia from LN clinical trials. Nevertheless, vigilance is required for serious or recurrent infections in LN, and for comorbid common variable immunodeficiency.

## References

[1] Lim E, Tao Y, White AJ, et al Hypogammaglobulinemia in pediatric systemic lupus erythematosus. Lupus 2013;22:1382–7. doi:10.1177/0961203313507990 24106215 10.1177/0961203313507990 PMC3840537

[2] Yap DY, Yung S, Ma MK, et al Serum immunoglobulin G level in patients with lupus nephritis and the effect of treatment with corticosteroids and mycophenolate mofetil. Lupus 2014;23:678–83. doi:10.1177/0961203314525248 24554708 10.1177/0961203314525248

[3] Quinti I, Soresina A, Guerra A, et al Effectiveness of immunoglobulin replacement therapy on clinical outcome in patients with primary antibody deficiencies: results from a multicenter prospective cohort study. J Clin Immunol 2011;31:315–22. doi:10.1007/s10875-011-9511-0 21365217 10.1007/s10875-011-9511-0

[4] Ginzler EM, Wax S, Rajeswaran A, et al Atacicept in combination with MMF and corticosteroids in lupus nephritis: results of a prematurely terminated trial. Arthritis Res Ther 2012;14:R33 doi:10.1186/ar3738 22325903 10.1186/ar3738 PMC3392829

[5] ACCESS Trial Group. Treatment of lupus nephritis with abatacept: the abatacept and cyclophosphamide combination efficacy and safety study. Arthritis Rheumatol 2014;66:3096–104. doi:10.1002/art.38790 25403681 10.1002/art.38790 PMC4528976

[6] Houssiau FA, Vasconcelos C, D'Cruz D, et al Immunosuppressive therapy in lupus nephritis: the euro-lupus nephritis trial, a randomized trial of low-dose versus high-dose intravenous cyclophosphamide. Arthritis Rheum 2002;46:2121–31. doi:10.1002/art.10461 12209517 10.1002/art.10461

[7] Furst DE Serum immunoglobulins and risk of infection: how low can you go? Semin Arthritis Rheum 2009;39:18–29. doi:10.1016/j.semarthrit.2008.05.002 18620738 10.1016/j.semarthrit.2008.05.002

[8] Marco H, Smith RM, Jones RB, et al The effect of rituximab therapy on immunoglobulin levels in patients with multisystem autoimmune disease. BMC Musculoskelet Disord 2014;15:178 doi:10.1186/1471-2474-15-178 24884562 10.1186/1471-2474-15-178 PMC4038057

[9] Roberts DM, Jones RB, Smith RM, et al Rituximab-associated hypogammaglobulinemia: incidence, predictors and outcomes in patients with multi-system autoimmune disease. J Autoimmun 2015;57:60–5. doi:10.1016/j.jaut.2014.11.009 25556904 10.1016/j.jaut.2014.11.009

[10] Roberts DM, Jones RB, Smith RM, et al Immunoglobulin G replacement for the treatment of infective complications of rituximab-associated hypogammaglobulinemia in autoimmune disease: a case series. J Autoimmun 2015;57:24–9. doi:10.1016/j.jaut.2014.11.004 25586449 10.1016/j.jaut.2014.11.004

[11] Venhoff N, Effelsberg NM, Salzer U, et al Impact of rituximab on immunoglobulin concentrations and B cell numbers after cyclophosphamide treatment in patients with ANCA-associated vasculitides. PLoS One 2012;7:e37626 doi:10.1371/journal.pone.0037626 22629432 10.1371/journal.pone.0037626 PMC3357389

[12] Besada E, Koldingsnes W, Nossent JC Long-term efficacy and safety of pre-emptive maintenance therapy with rituximab in granulomatosis with polyangiitis: results from a single centre. Rheumatology 2013;52:2041–7. doi:10.1093/rheumatology/ket257 23934313 10.1093/rheumatology/ket257

[13] Soybilgic A, Onel KB, Utset T, et al Safety and immunogenicity of the quadrivalent HPV vaccine in female systemic lupus erythematosus patients aged 12 to 26 years. Pediatr Rheumatol Online J 2013;11:29 doi:10.1186/1546-0096-11-29 23924237 10.1186/1546-0096-11-29 PMC3751269

[14] Fernández-Castro M, Mellor-Pita S, Citores MJ, et al Common variable immunodeficiency in systemic lupus erythematosus. Semin Arthritis Rheum 2007;36:238–45. doi:10.1016/j.semarthrit.2006.09.005 17276173 10.1016/j.semarthrit.2006.09.005

[15] Xiao X, Miao Q, Chang C, et al Common variable immunodeficiency and autoimmunity--an inconvenient truth. Autoimmun Rev 2014;13:858–64. doi:10.1016/j.autrev.2014.04.006 24747700 10.1016/j.autrev.2014.04.006

